# Supplementary material for: Navigability of Random Geometric Graphs in the Universe and Other Spacetimes
Source: Sci Rep. 2017 Aug 18;7:8699. doi: 10.1038/s41598-017-08872-4 (PMC5562713; doi:10.1038/s41598-017-08872-4)
Supplement: Supplementary file 1 — Supplementary Figures [file 41598_2017_8872_MOESM1_ESM.pdf]

Supplementary Information: Navigability of Random Geometric Graphs in the Universe and Other Spacetimes

William Cunningham,[1] Konstantin Zuev,[2] and Dmitri Krioukov[3]

[1] *Department of Physics, Northeastern University, 360 Huntington Ave., Boston, MA 02115, United States*

[2] *Department of Computing and Mathematical Sciences, California Institute of Technology, 1200 E. California Blvd., Pasadena, CA 91125, United States*

[3] *Department of Physics, Department of Mathematics, Department of Electrical & Computer Engineering, Northeastern University, 360 Huntington Ave., Boston, MA 02115, United States*

Supplementary Figures

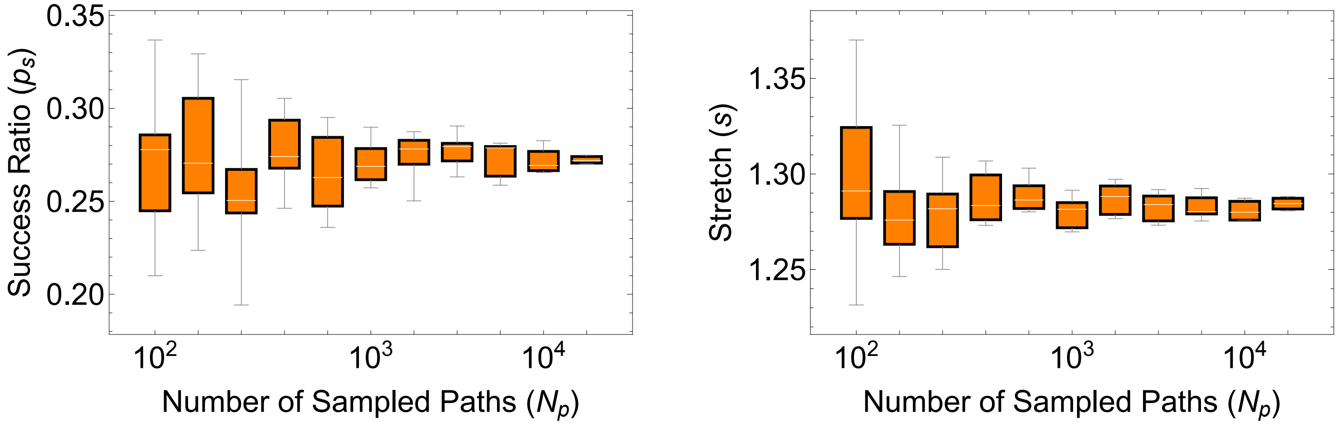

Figure S1: **Convergence of success ratio and stretch.** The boxplots summarize the distributions of the success ratio (a) and stretch (b) as functions of the number N_p of random source-destination node pairs sampled in 10 random geometric graphs (N_p pair samples in each graph) in the Einstein-de Sitter (dust) manifold with $\tau_0 = 4.64$, $\bar{k} = 10$, and $N = 2^{20}$. The orange boxes range from the first to third quartiles, while the bars are minima and maxima. The distributions stabilize at $N_p \ll N$.

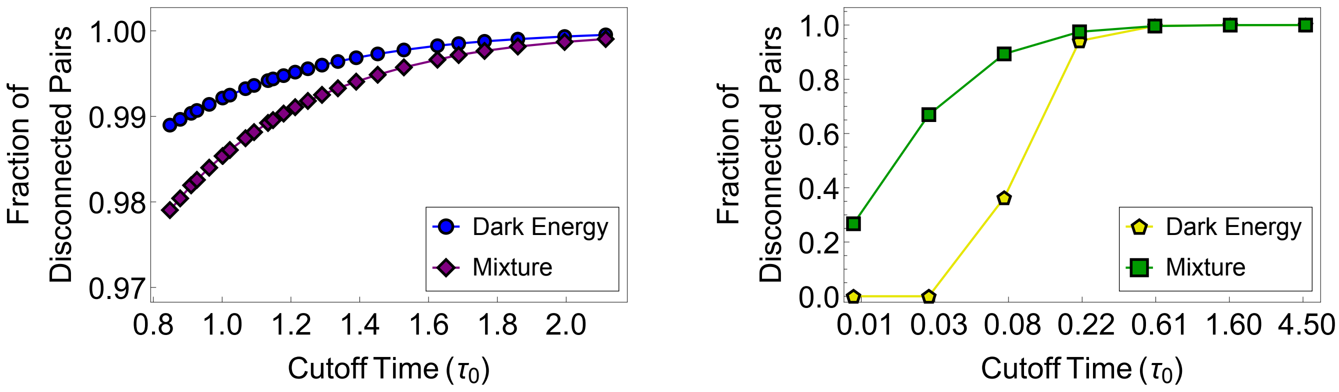

Figure S2: **Fraction of geodesically disconnected node pairs** in the graphs in Figs. 2-4 in the main text. Panels (a,b) correspond to the graphs in the de Sitter (dark energy) and mixed manifolds with $q = 60, \rho_0 = 6$ and $N = 2^{20}, \bar{k} = 10$, respectively. The graphs in the Einstein-de Sitter (dust) manifold have trivially no geodesically disconnected node pairs since the manifold is geodesically connected.

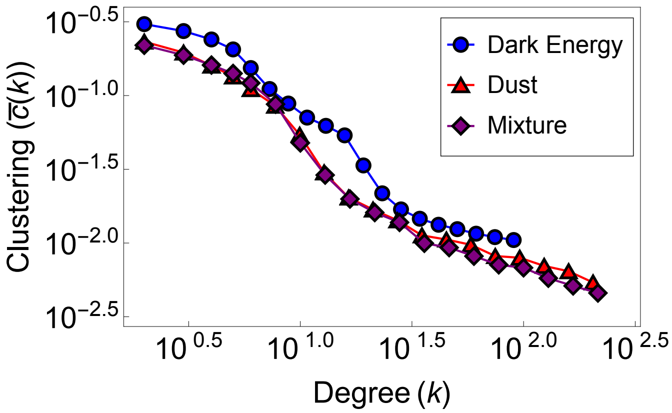

Figure S3: **Clustering in Lorentzian RGGs.** The figure shows the average clustering $\bar{c}(k)$ of nodes of degree k in random geometric graphs with $q = 60, \rho_0 = 6, \tau_0 = 0.84$ in the three studied manifolds. The mean clustering excluding nodes with $k = \{0, 1\}$ in the de Sitter, Einstein-de Sitter, and mixed manifolds are $\bar{c}_E = 0.145$, $\bar{c}_D = 0.164$, and $\bar{c}_M = 0.166$, respectively.

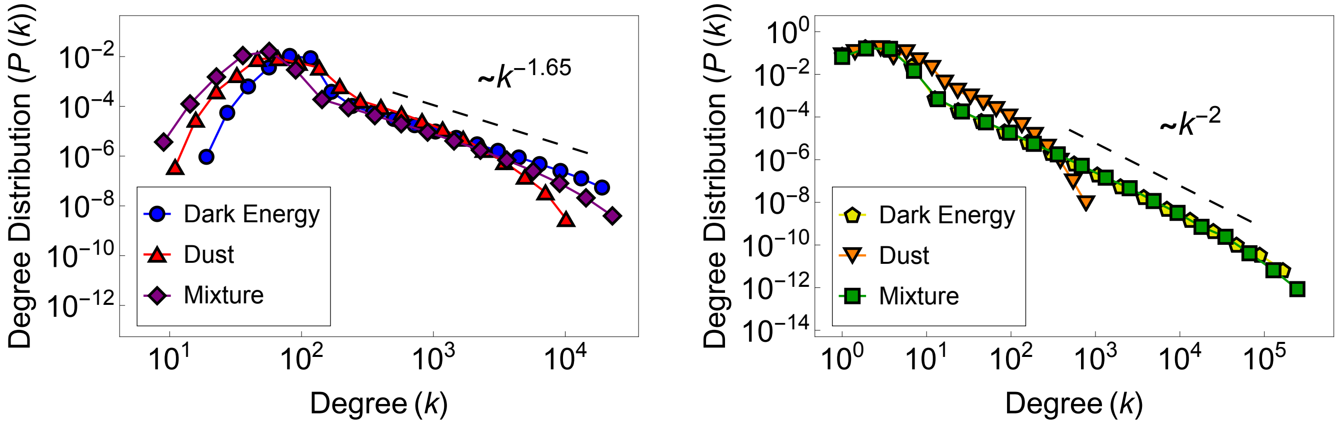

Figure S4: **Degree distribution in Lorentzian RGGs.** Panels (a) and (b) show the degree distribution in the random geometric graphs in the three considered manifolds in the constant-q and constant-$N, \bar{k}$ experiments, respectively, at the largest considered cut-off times τ_0. Specifically, in panel (a) $q = 60$, $\bar{k} = 130$, $N = 2518528$, $\tau_0 = 2.11$, and $\rho_0 = 6$, while in panel (b) $q = 0.564$, $\bar{k} = 10$, $N = 2^{20}$, $\tau_0 = 4.64$, and $\rho_0 = 1.68$.

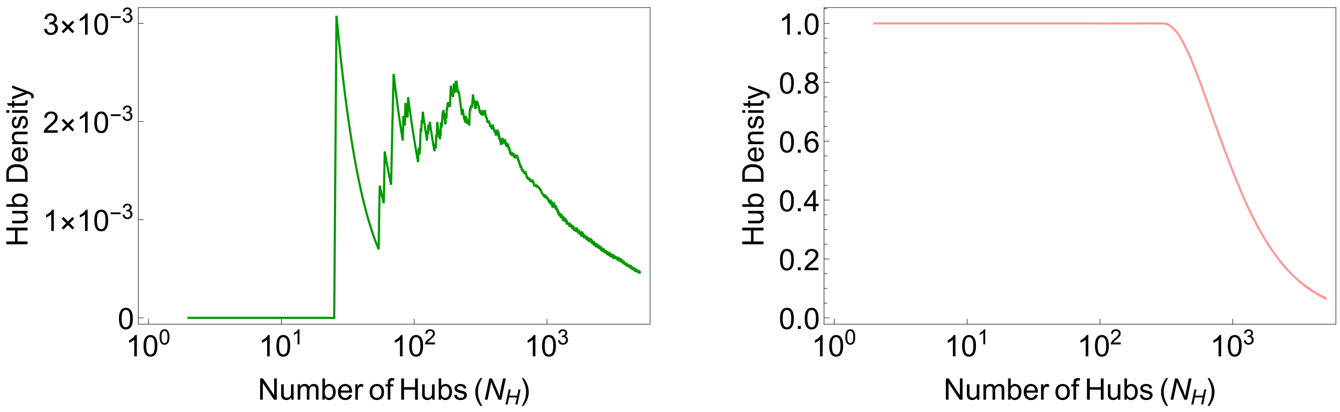

Figure S5: **Hub density in Lorentzian and hyperbolic random graphs.** The hub density is defined as the number of links among the N_H nodes with largest degrees, divided by the maximum possible number $\binom{N_H}{2}$ of such links. Panels (a,b) compare the hub density in two random graphs of the same size $N = 2^{20}$ and average degree $\bar{k} = 10$. Panel (a) shows the data for the mixed-content (M) Lorentzian manifold graph with $\rho_0 = 1.68$ and $\tau_0 = 4.64$, while panel (b) shows the same data for the hyperbolic graph generated using `http://named-data.github.io/Hyperbolic-Graph-Generator/` with parameters $N = 2^{20}$, $\bar{k} = 10$, $\gamma = 2$, and $T = 0$ (the resulting radial cutoff is $\rho_0 = 32.36$). There are exactly zero links between 25 largest-degree nodes in the Lorentzian graph, while the subgraph induced by the first 103 highest-degree nodes in the hyperbolic graph is the complete graph.

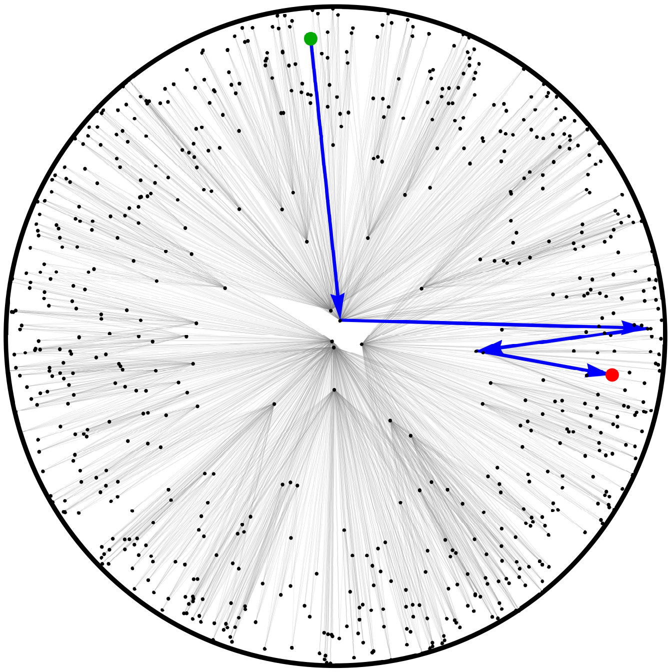

Figure S6: **A typical navigation path in a Lorentzian RGG.** The figure shows the greedy geometric routing navigation path from the spacelike-separated green source and red destination in the same graph as in Figure 1 in the main text. The greedy path, which is also the shortest (stretch-1) path in the graph, alternates between hubs and peripheral nodes. Any timelike-separated pairs of nodes are directly linked, resulting in trivial one-hop stretch-1 paths.
